# Acute Infectious Morbidity in Multiple Gestation

**DOI:** 10.1155/2015/173261

**Published:** 2015-01-05

**Authors:** Sarah K. Dotters-Katz, Emily Patel, Chad A. Grotegut, R. Phillips Heine

**Affiliations:** ^1^Division of Maternal-Fetal Medicine, Department of Obstetrics and Gynecology, University of North Carolina, Chapel Hill, NC 27599, USA; ^2^Division of Maternal-Fetal Medicine, Department of Obstetrics and Gynecology, Duke University, Durham, NC 27705, USA

## Abstract

*Objectives*. Physiologic and immunologic changes in pregnancy result in increased susceptibility to infection. These shifts are more pronounced in pregnancies complicated by multiple gestation. The objective of this study was to determine the association between multiple gestation and risk of infectious morbidity. *Study Design*. The Nationwide Inpatient Sample for the years 2008–2010 was used to identify pregnant women during admission for delivery with International Classification of Diseases codes. Logistic regression was used to compute odds ratios and 95% confidence intervals for demographic data, preexisting medical conditions, and acute medical and infectious complications for women with multiple versus singleton gestations. *Results*. Among women with multiple gestation, 38.4 per 1,000 women had an infectious complication compared to 12.8 per 1,000 women with singletons. The most significant infectious morbidity associated with multiple gestation was intestinal infections, pyelonephritis, influenza, and pneumonia. After controlling for confounding variables, infectious complications at delivery persisted for women with multiples, though the association was dependent on mode of delivery. *Conclusions*. Women with multiple gestations are at increased risk for infectious morbidity identified at the time of delivery. This association was diminished among women who had a cesarean suggesting that operative delivery is not responsible for this association.

## 1. Introduction

Many physiologic and immunologic changes occur during pregnancy. These changes are necessary for the development of the fetus. For example, elevated progesterone levels needed to support the pregnancy also lead to decreased ureteral peristalsis [1, 2]. This, coupled with ureteral compression by the gravid uterus, leads to increased susceptibility to infections in the urinary tract during pregnancy. Similarly, the adaptive immune response (including the T-cell and B-cells response) is decreased; therefore, the ability to form antibodies and generate a cell-mediated defense is dampened [2–4]. Because of this, there is an increased susceptibility to viral and fungal infections.

In pregnancies complicated by multiple gestation, these physiologic changes may be more pronounced. There are higher levels of progesterone in multiple gestations; therefore, the effects of progesterone may be increased [5]. Additionally, alterations to the immune response are amplified. Women with multiples exhibit an increase shift from T-helper 1 to T-helper 2 immunity as compared to singletons, further decreasing the activation of macrophages, B-cells, and CD8 T cells in this population [6]. This evidence suggests that in multiple gestation physiologic changes are more pronounced and immune response is dampened, both of which may increase the risk of infections in women pregnant with multiples. However, little data exist to substantiate this theory. Thus, the objective of this study was to determine the association between multiple gestation and risk of infectious morbidity.

## 2. Materials and Methods

### 2.1. Study Design

Discharge data from the Nationwide Inpatient Sample [7] from the Healthcare Cost and Utilization Project of the Agency for Healthcare Research and Quality (AHRQ) was obtained from 2008 to 2010. The NIS is the largest all-payer inpatient care database in the United States [8]. It contains information pertaining to more than 8 million annual hospital stays at more than 1,000 hospitals in over 42 states. The NIS stratifies hospitals based on ownership, bed size, teaching status, urban/rural location, and region. From each stratum, the NIS encompasses approximately 20% of United States hospitals, and the sampling frame comprises 90% of all US hospital discharges. Information within the NIS is similar to a discharge summary. Care is taken to protect the privacy of individual patients, physicians, and hospitals. National assessments are made via weighted estimates provided in the NIS.

The NIS database was queried for the years 2008–2010. All delivery-related discharges were identified, defined as a hospitalization, which included a delivery code (International Classification of Diseases, 9th Revision, Clinical Modification [ICD-9-CM] codes 74.x [except 74.91] for cesarean section and V27, 72.x, 73.x, and 650 for general delivery codes [not utilized to specify mode of delivery]). Diagnosis-related group (DRG) codes of 767, 768, 774, and 775 were also used to identify vaginal deliveries, while DRG codes of 765 and 766 were used to identify cesarean deliveries. Women carrying a multiple gestation were identified using the ICD-9-CM codes 651.0x, 651.1x, 651.2x, 651.8x, 651.9x, and V27.2-V27.4. Medical comorbidities and nonobstetric infections were identified using the ICD-9-CM code for a particular condition in pregnancy and the general ICD-9-CM codes were used (see Supplemental Table 1 in Supplementary Material available online at http://dx.doi.org/10.1155/2015/173261 for list of ICD-9-CM codes used). Obstetric complications were identified similarly.

### 2.2. Statistical Analysis

Once delivery-related admissions were identified, logistic regression analysis was used to calculate odds ratios with 95% confidence intervals for demographic data, medical conditions prior to pregnancy, obstetric outcomes, and nonobstetric infections in patients with multiple compared to women with singleton gestation. A multivariable logistic regression model was then created, controlling age, race/ethnicity, insurance status, length of stay, hypertension, diabetes, gestational diabetes, asthma, HIV, systemic lupus erythematosus, collagen vascular disease/rheumatoid arthritis, anemia, thrombocytopenia, and preterm labor. We elected to control medical conditions likely affecting the risk for infectious morbidity. Because mode of delivery was identified as an interaction term, in the final model, women who had vaginal deliveries and those who underwent cesarean delivery were analyzed separately. A *P* value of less than 0.05 was considered statistically significant. SAS version 9.3 (SAS Institute Inc., Cary, NC) and GraphPad Prism 6.0 for Macintosh (GraphPad Software, San Diego, CA) were used to complete the analyses. This study protocol was reviewed and approved by the Duke University Medical Center Institutional Review Board as exempt research.

## 3. Results

Between 2008 and 2010, there were 12,524,118 delivery-related discharges. Of these, there were 266,751 pregnancies with multiple gestation. The overall multiple gestation rate during this period was 2.1 per 100 deliveries.

Table 1 outlines demographic information in patients with singleton compared to multiple gestation. Women with multiple gestation were more likely to be older and to have private insurance. The length of stay was also longer in the population with multiple gestation. Women with multiple gestation were also less likely to reside within a ZIP code with a median household income in the lowest quartile.

Women with multiple gestation were more likely to have asthma, endocrine disorders, and autoimmune disorders (Table 2). Women with multiples were also more likely to have chronic hypertension and chronic renal disease. Hematologic disorders, such as anemia, thrombocytopenia, and thrombophilias, were also more common among women with multiple gestation. In contrast, tobacco and drug use were more common in women with singletons.

Obstetric complications present at the time of discharge for delivery are listed in Table 3. Gestational diabetes and preeclampsia were more common among women with multiple gestation. Women with multiple gestation were more likely to have preterm labor, as would be expected. In contrast, operative vaginal delivery and chorioamnionitis were less common in women with multiples. However, cesarean delivery and endometritis were more common in pregnancies complicated by multiple gestation.

Table 4 describes nonobstetric infections present at delivery in singleton and multiple gestations. Among women with multiple gestation, 38.4 per 1,000 women had an infectious complication compared to 12.8 per 1,000 women with singleton gestation (OR 3.12, CI 3.05, 3.18 for composite infection). Minor infections, such as otitis media, pharyngitis, sinusitis, and cellulitis, were all more common in women with multiple gestation. Similarly, more severe infections were also all more common in this population. Women with multiple gestation were 3.2 times more likely to have bacteremia and 2.9 times more likely to have sepsis. Pulmonary infections such as pneumonia and influenza occurred 3.6 and 3.5 times more, respectively, in women with multiples. Pyelonephritis and urinary tract infections were also approximately three times as common in women with multiples. Overall, the composite infection risk for any nonobstetric infection in women with multiple gestation was 3.1 times higher than for women with singletons.

A multivariable logistic regression model for moderate to severe infections among multiples compared to singletons by mode of delivery, while controlling age, race/ethnicity, insurance status, length of hospital stay, chronic hypertension, gestational and preexisting diabetes, asthma, HIV, SLE, collagen vascular disease/rheumatoid arthritis, anemia, thrombocytopenia, and preterm labor is described in Table 5. Among women who delivered vaginally, the odds of pneumonia were 3.3 times higher in women with multiple gestations compared to singletons. However, among those delivering by cesarean, the odds of pneumonia were lower in women with multiple compared to women with singleton gestations (OR 0.66, 95% CI 0.58, 0.74). Similarly, the odds of pyelonephritis were 5.5 times higher among multiples compared to singletons that delivered vaginally. Yet among those delivering by cesarean, women with multiple gestation were less likely to have pyelonephritis compared to those with singletons (OR 0.70, 95% CI 0.59, 0.83). Women with multiples who delivered vaginally had fivefold increased odds for appendicitis compared to women with singletons who delivered vaginally. Women with multiples who delivered by cesarean were less likely to have appendicitis compared to women with a singleton gestation delivering via cesarean (OR 0.17, 95% CI 0.09, 0.29). There was no difference among multiples who delivered by cesarean in rates of influenza or intestinal infections as compared to singletons (OR 0.92, 95% CI 0.69, 1.22 and OR 1.21, 95% CI 0.97, 1.49). Overall, the risk of infectious morbidity among women with multiple gestation was increased in the vaginal delivery group and decreased in the cesarean delivery subset.

Of the 266,751 deliveries to multiples, 16,517 (6.2%) included specific codes for triplet or quadruplet gestation. As the physiology of pregnancies complicated by higher order multiples may be different from that of twin gestation, the multivariable logistic regression analysis was repeated. This analysis excluded women with specific codes for triplet or quadruplet gestation (651.1x, 651.2x) and had similar results to the analysis which included all women carrying a multiple gestation (Supplemental Table 2).

## 4. Conclusions

Nonobstetric infections are a common cause of morbidity and occasionally mortality throughout pregnancy [4, 9–12]. This study identified that in women with multiple gestation, there is an increased risk of nonobstetric infections when compared to women carrying singletons. Specifically, the risks of pyelonephritis, influenza, and pneumonia were all significantly elevated. After controlling age, race/ethnicity, insurance status, length of hospital stay, chronic hypertension, gestational and preexisting diabetes, and preterm labor, women with multiple gestation who delivered vaginally remained at an increased risk for these nonobstetric infections, while those who delivered by cesarean section were at a decreased risk when compared to women with singletons.

Various authors have shown increased incidence and increased morbidity associated with pulmonary infection during pregnancy [5, 10, 12, 13]. The increased infection severity has been attributed to the net decrease in expiratory reserve volume, functional residual capacity, and residual volume [5, 14]. Siston et al. found these risks to be true with the H1N1 pandemic in 2009, during which pregnant women comprised 5% of all H1N1 related deaths, but only represented 1% of the population [15]. Similarly, other authors have seen increased hospitalization and increased ICU admissions attributable to yearly influenza outbreaks [12, 16]. Based on our NIS data, women with multiple gestation are 3.5 times more likely to develop influenza or pneumonia than their singleton counterparts. This is even more striking when one considers that mortality from these infections is highest in the third trimester (the height of maternal physiologic alterations) and considering that these changes occur earlier in pregnancies complicated by multiple gestation [5, 12, 13]. Given that yearly influenza and even some bacterial pneumonias are preventable with vaccination, the importance of vaccination in this population cannot be understated.

Urologic complications of pregnancy are more common than pulmonary complications and have the ability to be as severe. Progesterone levels are significantly higher in multiple pregnancies [2, 5]. Additionally, the size of the renal collecting systems is also increased when compared to singleton gestation [5, 14]. Finally, the compression of the ureters by the gravid uterus occurs earlier in gestation with multiples [2]. All of these likely contribute to the increased risk of urinary tract infection as well as pyelonephritis in multiple gestation. Pyelonephritis in pregnancy has been shown by many authors to be associated with sepsis, septic shock, acute respiratory distress syndrome (ARDS), and ICU admission [9, 17]. Given the increased risk of urinary tract infections and pyelonephritis in multiple gestations, compounded with the alterations in pulmonary pathology, the risk of complications such as sepsis, ARDS, and ICU admission is likely increased in pregnancies with multiple gestation. Further research is needed to confirm this.

This data has also brought to light an interesting difference regarding infection and mode of delivery. Women with multiple gestation who undergo vaginal delivery have a threefold risk of infection compared to their singleton counterparts. In contrast, the composite infection risk is decreased in women with multiples who undergo cesarean section. It is likely that many patients with multiple gestation will undergo elective delivery near term and a serious infectious morbidity would preclude providers from scheduling the procedure until the infection has resolved. Another possible explanation is that a more severe infection may incite labor, leading to rapid vaginal delivery as well as a reluctance among providers to perform a cesarean in the setting of an active infection and a potentially unstable mother. This data does help dispel the assumption that cesarean section is associated with increased nonobstetric infectious morbidity among multiple gestations. However, any further conclusions on this finding would be speculative, and more research is needed to further explain these findings.

Though a valuable resource, the NIS database does impose certain limitations on this study. First, due to inherent limitations within the NIS, it is not possible to determine the gestational age at delivery. Similarly, we are not able to determine which patients labored and subsequently had a cesarean versus those that had a planned cesarean. However, it could be surmised that a patient who labors and then undergoes cesarean delivery is at higher risk for infection, not a lower risk as was noted in this data. Additionally, there is no way to verify the timing of infection relative to delivery. We cannot know if the patient had an infection with subsequent delivery or if the infection occurred postpartum during the delivery admission as only infections at delivery admission were included. Therefore, we cannot determine if there is causal relationship between mode of delivery and infection or between multiple gestation and infection. Though the multivariable logistic regression model attempted to control factors that might predispose to infection, it is not possible to control all confounders. This is another limitation of the study.

Another limitation to our study is the use of ICD-9 codes to identify patients and outcomes. However, Yasmeen et al. recently inspected a variety of obstetric discharge databases for reliability and accuracy and found them to be satisfactory sources of information [18]. Thus, despite the inherent limitations, the NIS is a valuable resource for the study of rare disorders and uncommon outcomes of which a study from a single center would be nearly impossible.

This dataset does bring to light the increased infection risk associated with multiple gestation. The increased risks of urologic and pulmonary infections are specifically notable, as these are infections, which may be preventable if caught early (urologic) or with vaccines (pulmonary). Future research is needed to better understand the role of mode of delivery on infectious risk in this cohort. However, even as an association, the increased infection risk for women with multiple gestation is significant.

## Supplementary Material

Supplementary Table 1. describes the ICD-9 codes that were used to identify the pre-existing medical conditions, medical events, and obstetric complications in the NIS 2008 – 2010.
Supplementary Table 2. details the multivariable logistic regression analysis by mode of delivery for infectious outcomes among women with multiple gestations, excluding triplets and quadruplets, compared to women with singleton gestations. The model controls for age, race/ethnicity, insurance status, length of hospital stay, chronic hypertension, gestational diabetes, diabetes, asthma, HIV, systemic lupus erythematosus, collagen vascular disease/rheumatoid arthritis, anemia, thrombocytopenia, and preterm labor.

## Figures and Tables

**Table 1:** Demographic data during hospital discharges for delivery among pregnant women with multiple gestation compared to women with singleton gestation, Nationwide Inpatient Sample for years 2008–2010 (12,524,118 deliveries).

	Multiple gestation *n* = 266,751	Singleton gestation *n* = 12,257,367	OR (95% CI)	*P* value
Race/ethnicity, *n* (%)				
Caucasian	131,828 (49.4)	5,402,164 (44.1)	1.0	—
African American	36,964 (13.9)	1,453,880 (11.9)	1.04 (1.03, 1.05)	<0.0001
Hispanic	33,546 (12.6)	2,371,751 (19.3)	0.58 (0.57, 0.59)	<0.0001
Other	21,887 (8.2)	1,132,263 (9.2)	0.79 (0.78, 0.81)	<0.0001
Missing	42,526 (15.9)	1,897,310 (15.5)	—	—
Age, yrs^a^	29.8 ± 139	27.6 ± 13.7	—	<0.0001
Private insurance, *n* (%)	162,185 (60.8)	6,055,139 (49.4)	1.56 (1.55, 1.57)	<0.0001
Median house income in ZIP code of lowest quartile, *n* (%)^b^	61,353 (23.0)	3,309,489 (27.0)	0.73 (0.72, 0.73)	<0.0001
LOS, days^c^	3 (2, 4)	2 (2, 3)	—	<0.0001

Total charges, $^c^	14,730 (9477, 23,228)	9953 (6778, 14,965)	—	<0.0001

^a^Values are mean ± SD.

^b^Median house income in ZIP code of lowest quartile defined as median income in subject's ZIP code $1–$38,999.

^c^Values are median (quartile).

**Table 2:** Preexisting medical conditions present during hospital discharges for delivery among pregnant women with multiple gestation compared to women with singleton gestation, Nationwide Inpatient Sample for years 2008–2010 (12,524,118 deliveries).

Condition, *n* (%)	Multiple gestation *n* = 266,751	Singleton gestation *n* = 12,257,367	OR (95% CI)	*P* value
Pulmonary disease				
Asthma	10,347 (3.9)	394,749 (3.2)	1.21 (1.19, 1.24)	<0.0001
Endocrine disorders				
Diabetes (nongestational)	3242 (1.2)	127,728 (1.0)	1.17 (1.13, 1.21)	<0.0001
Thyroid disorder	11,533 (4.3)	286,009 (2.3)	1.89 (1.86, 1.93)	<0.0001
Autoimmune disorders				
Systemic lupus erythematosus	420 (0.16)	13,930 (0.11)	1.39 (1.26, 1.53)	<0.0001
Collagen vascular disease/rheumatoid arthritis	469 (0.18)	14,932 (0.12)	1.44 (1.32, 1.58)	<0.0001
HIV	75 (0.028)	3541 (0.029)	0.98 (0.77, 1.22)	0.85
Hematologic disorders				
Thrombophilia/APS	2609 (1.0)	61,908 (0.50)	1.95 (1.87, 2.02)	<0.0001
Anemia	47,106 (17.7)	1,308,237 (10.7)	1.80 (1.78, 1.81)	<0.0001
Thrombocytopenia	5439 (2.0)	108,169 (0.9)	2.34 (2.27, 2.40)	<0.0001
Drugs/alcohol/tobacco				
Drug use	3029 (1.1)	159,237 (1.3)	0.87 (0.84, 0.90)	<0.0001
Alcohol use	258 (0.10)	13,392 (0.11)	0.89 (0.78, 1.00)	0.056
Tobacco	14,399 (5.4)	772,584 (6.3)	0.85 (0.83, 0.86)	<0.0001
Chronic hypertension/renal failure				
Chronic hypertension	8040 (3.0)	236,910 (1.9)	1.58 (1.54, 1.61)	<0.0001
Chronic renal failure	198 (0.07)	4719 (0.04)	1.93 (1.66, 2.21)	<0.0001

APS = antiphospholipid antibody syndrome.

**Table 3:** Obstetric events present during hospital discharges for delivery among pregnant women with multiple gestation compared to women with singleton gestation, Nationwide Inpatient Sample for years 2008–2010 (12,524,118 deliveries).

Condition, *n* (%)	Multiple gestation *n* = 266,751	Singleton gestation *n* = 12,257,367	OR (95% CI)	*P* value
Cesarean delivery	156,025 (58.5)	3,885,073 (31.7)	3.04 (3.01, 3.06)	<0.0001
Operative vaginal delivery	9773 (3.7)	782,470 (6.4)	0.56 (0.55, 0.57)	<0.0001
Gestational diabetes	20,495 (7.7)	688,577 (5.6)	1.40 (1.38, 1.42)	<0.0001
Preeclampsia	42,031 (15.8)	881,432 (7.2)	2.42 (2.39, 2.44)	<0.0001
Preterm labor	129,281 (48.5)	889,044 (7.2)	12.03 (11.93, 12.12)	<0.0001
Fetal growth restriction	19,968 (7.5)	251,510 (2.0)	3.86 (3.80, 3.92)	<0.0001
Fetal demise	1999 (0.7)	49,076 (0.4)	1.88 (1.80, 1.96)	<0.0001
Placental abruption	4900 (1.8)	129,870 (1.1)	1.75 (1.70, 1.80)	<0.0001
Postpartum hemorrhage	11,413 (4.3)	308,049 (2.5)	1.73 (1.70, 1.77)	<0.0001
Chorioamnionitis	5460 (2.0)	318,016 (2.6)	0.78 (0.76, 0.81)	<0.0001
Endometritis	10,178 (3.8)	160,446 (1.3)	3.00 (2.93, 3.05)	<0.0001

**Table 4:** Acute infections present during hospital discharges for delivery among pregnant women with multiple gestation compared to women with singleton gestation, Nationwide Inpatient Sample for years 2008–2010 (12,524,118 deliveries).

Condition, *n* (%)	Multiple gestation *n* = 266,751	Singleton gestation *n* = 12,257,367	OR (95% CI)	*P* value
Generalized infections				
Sepsis	368 (0.14)	5937 (0.05)	2.85 (2.56, 3.16)	<0.0001
Bacteremia	124 (0.05)	1767 (0.01)	3.23 (2.68, 3.85)	<0.0001
Neurologic				
Meningitis	5 (0.002)	74 (0.0006)	3.31 (1.20, 7.25)	0.008
Ear, nose, and throat				
Otitis media	106 (0.04)	2040 (0.02)	2.38 (1.96, 2.89)	<0.0001
Pharyngitis	147 (0.05)	3959 (0.03)	1.70 (1.45, 2.01)	<0.0001
Sinusitis	256 (0.10)	5380 (0.04)	2.19 (1.93, 2.48)	<0.0001
Pulmonary				
Pneumonia	864 (0.3)	11,026 (0.09)	3.61 (3.37, 3.87)	<0.0001
Influenza	297 (0.10)	3925 (0.03)	3.48 (3.09, 3.91)	<0.0001
Gastrointestinal				
Gastrointestinal infections^a^	450 (0.17)	3322 (0.027)	6.23 (5.64, 6.87)	<0.0001
Appendicitis	134 (0.05)	2239 (0.018)	2.76 (2.32, 3.29)	<0.0001
Urologic				
Urinary tract infections	5515 (2.1)	85,488 (0.70)	3.01 (2.93, 3.09)	<0.0001
Pyelonephritis	1392 (0.5)	19,280 (0.2)	3.33 (3.15, 3.51)	<0.0001
Skin/dermatology				
Cellulitis/abscess skin	907 (0.34)	15,452 (0.13)	2.70 (2.53, 2.89)	<0.0001
MRSA infection (unspec. site)	65 (0.02)	2290 (0.019)	1.31 (1.02, 1.67)	0.031

Composite Infection	10,210 (3.84)	156,613 (1.28)	3.12 (3.05, 3.18)	<0.0001

^a^Cholera, typhoid, salmonella, shigella, amebiasis, and intestinal protozoal diseases.

**Table 5:** Multivariable logistic regression analysis by mode of delivery for the listed infectious outcomes among women with multiple gestations compared to women with singleton gestations while controlling age, race/ethnicity, insurance status, length of hospital stay, chronic hypertension, gestational diabetes, diabetes, asthma, HIV, systemic lupus erythematosus [19], collagen vascular disease, anemia, thrombocytopenia, and preterm labor.

	Cesarean delivery		Vaginal delivery	
	Adjusted OR	*P* value	Adjusted OR	*P* value
	(95% CI)		(95% CI)	
Sepsis	0.38 (0.31, 0.46)	<0.0001	1.52 (1.26, 1.82)	<0.0001
Pneumonia	0.66 (0.58, 0.74)	<0.0001	3.26 (2.91, 3.65)	<0.0001
Influenza	0.92 (0.69, 1.22)	0.59	3.83 (3.22, 4.51)	<0.0001
Intestinal infectious diseases	1.21 (0.97, 1.49)	0.089	5.77 (4.97, 6.68)	<0.0001
Appendicitis	0.17 (0.09, 0.29)	<0.0001	5.03 (4.09, 6.13)	<0.0001
Urinary tract infections	0.85 (0.81, 0.90)	<0.0001	2.29 (2.20, 2.39)	<0.0001
Pyelonephritis	0.70 (0.59, 0.83)	0.0002	5.50 (5.13, 5.90)	<0.0001

Composite infection	0.83 (0.80, 0.86)	<0.0001	2.98 (2.89, 3.08)	<0.0001
