# Supplementary material for: Acute Infectious Morbidity in Multiple Gestation
Source: Infect Dis Obstet Gynecol. 2015 Jan 5;2015:173261. doi: 10.1155/2015/173261 (PMC4313678; doi:10.1155/2015/173261)
Supplement: Supplementary file 1 — Supplementary Table 1. describes the ICD-9 codes that were used to identify the pre-existing medical conditions, medical events, and obstetric complications in the NIS 2008 – 2010. Supplementary Table 2. details the multivariable logistic regression analysis by mode of delivery for infectious outcomes among women with multiple gestations, excluding triplets and quadruplets, compared to women with singleton gestations. The model controls for age, race/ethnicity, insurance status, length of hospital stay, chronic hypertension, gestational diabetes, diabetes, asthma, HIV, systemic lupus erythematosus, collagen vascular disease/rheumatoid arthritis, anemia, thrombocytopenia, and preterm labor. [file 173261.f1.pdf]

Table S1. ICD-9 codes for pre-existing medical conditions, medical events, and obstetric complications utilized to identify cases in the NIS 2008 – 2010.

Medical Condition	ICD-9 codes
**Pulmonary Disease**	
Asthma	493.x
**Endocrine**	
Diabetes (non-gestational)	249.x, 250.x, 648.0x
Thyroid disease	240.x-246.x, 648.1x
**Autoimmune**	
Systemic lupus erythema.	710.0, 695.4, 583.8
Rheumatoid arthritis/ collagen vascular disease	701.0, 710, 710.1x-710.9x, 714.x, 720.x, 725
**Hematologic**	
Thrombophilia (includes history of thrombosis and antiphospholipid syndrome [APS])	273.8, 286.53, 286.9, 289.81, 289.82, V12.51
Anemia	648.2x, 280.x, 285.x
Thrombocytopenia	287.3x, 287.4x, 287.5x
**Drug/Alcohol/Tobacco**	
Drug use	292.x, 304.x, 305.2x-305.9x, 655.5x, 760.70, 760.72-760.75, 779.5, 965.0x, V65.42
Alcohol use	291.x, 303.x, 305.0x, 760.71, 980.0x
Smoking	305.1, V15.82, 649.0x
**Chronic hypertension/renal failure**	
Chronic Hypertension	401.x-405.x, 437.2, 642.0x-642.2x
Chronic Renal Failure	585.x, 792.5, V42.0, V45.1, V56.x
**Event or Condition**	
**Infections**	
Sepsis	038.x, 790.7
Bacteremia	790.7
Meningitis	320.x, 321-321.8, 322.x

Otitis media	382.x
Pharyngitis	462, 034.0
Sinusitis	461.x
Pneumonia	480-486
Influenza	487.x-488.x
Gastrointestinal infections	001-009.3
Appendicitis	540-542
Urinary Tract Infections	599.0
Pyelonephritis	590.x
Cellulitis/skin abscess	681.x
MRSA infection(unspecified site)	482.42
**Obstetric Events**	
Multiple Gestation	651.x, 652.6x, V27.2-V27.7
Operative vaginal delivery	Procedure codes: 720-724, 726, 727-727.9
Gestational diabetes	648.8x
Preeclampsia, eclampsia or gestational hypertension	642.3x-642.7x
Preterm labor	644.x
Placental abruption	641.2x
Fetal growth restriction	656.5x
Intrauterine fetal death	656.4x
Placenta previa	641.0-641.1x
Postpartum hemorrhage	666-666.2x
Chorioamnionitis	658.4x, 659.2x

Supplemental Table 2: Multivariable logistic regression analysis by mode of delivery for the listed infectious outcomes among women with multiple gestations, excluding those with specific codes for triplets and quadruplets, compared to women with singleton gestations while controlling for *age, race/ethnicity, insurance status, length of hospital stay, chronic hypertension, gestational diabetes, diabetes, asthma, HIV, systemic lupus erythematosus [19], collagen vascular disease/rheumatoid arthritis, anemia, thrombocytopenia, and preterm labor.* There were 250,234 multiple gestation deliveries (excluding triplets and quadruplets) and 12,257,367 deliveries to women with a singleton.

	**Cesarean delivery**		**Vaginal delivery**	
	**Adjusted OR (95% CI)**	**p-value**	**Adjusted OR (95% CI)**	**p-value**
Sepsis	0.33 (0.26, 0.41)	<0.0001	1.29 (1.05, 1.57)	0.0124
Pneumonia	0.67 (0.59, 0.76)	<0.0001	3.14 (2.78, 3.53)	<0.0001
Influenza	0.98 (0.73, 1.29)	0.90	3.79 (3.17, 4.50)	<0.0001
Intestinal infectious diseases	1.24 (0.99, 1.53)	0.055	5.54 (4.74, 6.44)	<0.0001
Appendicitis	0.18 (0.09, 0.31)	<0.0001	4.75 (3.81, 5.86)	<0.0001
Urinary Tract infections	0.85 (0.81, 0.90)	<0.0001	2.28 (2.18, 2.38)	<0.0001
Pyelonephritis	0.75 (0.63, 0.88)	0.0006	5.85 (5.45, 6.28)	<0.0001
**Composite Infection**	0.84 (0.80, 0.87)	<0.0001	2.97 (2.88, 3.07)	<0.0001
